# M2 macrophage derived HMOX1 defines chronic rhinosinusitis with nasal polyps

**DOI:** 10.1002/clt2.70014

**Published:** 2024-12-07

**Authors:** Enhao Wang, Yanghe Hao, Jing Song, Jing Yuan, Yu Hong, Ying Li, Yang Wang, Chengshuo Wang, Ming Wang, Luo Zhang

**Affiliations:** ^1^ Department of Otolaryngology Head and Neck Surgery Beijing TongRen Hospital Capital Medical University Beijing China; ^2^ Beijing Institute of Otolaryngology Beijing Laboratory of Allergic Diseases Beijing Key Laboratory of Nasal Diseases Key Laboratory of Otolaryngology Head and Neck Surgery Ministry of Education Capital Medical University Beijing China; ^3^ Research Unit of Diagnosis and Treatment of Chronic Nasal Diseases Chinese Academy of Medical Sciences Beijing China; ^4^ Department of Allergy Beijing TongRen Hospital Capital Medical University Beijing China

**Keywords:** CD163, chronic rhinosinusitis with nasal polyps, diagnostic signature, HMOX1, M2 macrophages

## Abstract

**Background:**

Molecular signatures of chronic rhinosinusitis with nasal polyps (CRSwNP) related to macrophages remain unclear. This study aimed to develop a macrophage‐associated diagnostic signature for CRSwNP.

**Methods:**

Transcriptome data from 54 patients with CRSwNP and 37 healthy controls across GSE136825, GSE36830, and GSE72713 were used to identify differentially expressed genes (DEGs) between two groups. Gene Set Enrichment Analysis and Weighted Gene Co‐Expression Network Analysis pinpointed crucial pathways and gene clusters. A diagnostic model was created from these analyses and receiver operating characteristic curve (ROC), and further validated in our transcriptome data from 29 samples. Immune cell infiltration analysis was performed and linked those diagnostic genes to macrophages and verified by single‐cell RNA sequencing data. Immunofluorescence co‐staining of CD163 and HMOX1 was performed in nasal tissues. Mouse bone marrow‐derived macrophage (BMDMs) cultures were used in functional experiments. Correlations between the expression of HMOX1 and eotaxin genes were investigated.

**Results:**

DEGs of CRSwNP versus control group were enriched in the INTERLEUKIN_4_AND_13_SIGNALING pathways. A four‐gene diagnostic model (HMOX1, ALOX5, F13A1 and ITGB2) was developed and demonstrated high diagnostic precision with an area under ROC curve of 0.980 for training dataset and 0.895 for test dataset. M2 macrophage presence and HMOX1 expression significantly correlated with CRSwNP (*p* < 0.001). Single‐cell RNA sequencing data underscored the altered cellular composition in CRSwNP, with HMOX1 notably expressed in M2 macrophages. Immunofluorescence staining highlighted the increased infiltration of CD163+ M2 macrophages in nasal mucosa samples of eosinophilic CRSwNP, which correlated with HMOX1 protein levels (*p* < 0.05). The HMOX1 inhibitor zinc protoporphyrin reduced the ratio of CD163 + HMOX1 + M2 macrophages in mouse BMDM cultures (*p* < 0.05). HMOX1 expression showed a strong positive correlation with the expression of eotaxin genes (CCL11, CCL24, and CCL26; *p* < 0.05 respectively).

**Conclusion:**

M2 macrophage‐derived HMOX1 can be used as an innovative diagnostic signature for CRSwNP, which might be a potential regulator of eosinophilic inflammation.

## 1 INTRODUCTION

Chronic rhinosinusitis with nasal polyps (CRSwNP) is a chronic inflammation of the nasal mucosa and sinuses. Symptoms of the disease include anterior or posterior nasal leakage, nasal congestion, olfactory dysfunction and/or facial pressure or pain that last for more than 12 weeks^1^ CRSwNP affects 1%–4% of the population and imposes significant economic costs.^2^, ^3^ The diagnosis of CRSwNP is often based on clinical manifestations, diagnostic examinations, and intrinsic type of disease based on biological markers.^4^ Several researches have shown that different biological markers could be used to diagnose CRSwNP. For instance, the presence of eosinophils, identified through the eosinophil cationic protein (ECP), is commonly associated with CRSwNP.^5^ Additionally, studies have identified increased levels of periostin, a marker linked to tissue remodeling, as a reliable indicator of disease severity in CRSwNP patients, suggesting its potential use in clinical diagnosis.^6^

The cellular and molecular mechanisms underlying the pathogenesis of CRSwNP are not fully understood.^7^, ^8^ Defects in the nasal epithelial barrier, increased exposure to pathogenic and colonizing bacteria, and dysregulation of the host immune system are thought to contribute to the disease.^9^, ^10^ Recent studies indicated that macrophages play an important role in the pathogenesis of CRSwNP.^10^ For instance, ALOX15+ macrophages contributed to the type 2 inflammation of CRSwNP by secreting chemokines that recruited eosinophils and Th2 cells.^11^ Moreover, Axl‐driven M2 macrophage polarization exacerbates disease severity in CRSwNP patients and leads to postoperative recurrence.^12^ Thus, the role of macrophages in CRSwNP needs to be further explored. The aim of this study was to construct a macrophage‐associated diagnostic signature for CRSwNP, and explore its potential roles.

## 2 MATERIALS AND METHODS

### 2.1 Subjects

Nasal mucosal samples of middle turbinate were collected from patients with CRSwNP and healthy individuals undergoing septoplasty for anatomical reasons at the Rhinology Department of Beijing TongRen Hospital. The diagnosis of CRSwNP adhered to the European Position Paper on Rhinosinusitis and Nasal Polyps 2020 guidelines.^3^ Exclusion criteria included severe systemic diseases, immunodeficiency, fungal sinusitis, and pregnancy. Participants had not received systemic or topical corticosteroids 4 weeks prior to the study. Furthermore, patients receiving systemic treatments, including biologicals or other immunomodulatory drugs, were explicitly excluded from the study. Nasal polyp tissues from CRSwNP patients and nasal mucosa tissues from healthy controls were surgically obtained for biopsy. The collected nasal mucosal samples were used for immunofluorescence staining and transcriptome analysis, which was used as the TongRen test dataset; clinic characteristics of participants were introduced previously.^13^ This study received approval from the ethics committee of Beijing TongRen Hospital.

### 2.2 Transcriptome expression matrix for training set

The training set data for the diagnostic model were obtained from the GEO database by searching with the keywords “(Chronic[All Fields] AND Rhinosinusitis[All Fields] AND (‘nasal polyps’[MeSH Terms] OR Nasal Polyps[All] Fields])) AND “*Homo sapiens*”[porgn]”. The datasets of GSE136825, GSE36830 and GSE72713 were collected and merged. Transcriptome expression matrices of 37 healthy control samples and 54 CRSwNP samples were obtained. All the sample information of the training dataset and test dataset are shown in Table S1.^14^, ^15^, ^16^

### 2.3 Gene set enrichment analysis (GSEA)

GSEA is a computational method used to determine whether a predefined set of genes shows statistically significant and consistent differences between two biological states, such as phenotypes. In this study, the GSEA and C2 collection: curated gene sets module of the Molecular Signatures Database (MSigDB) was used for analysis. Permutation type is phenotype. The number of permutations was set to 1000.

### 2.4 Weighted gene co‐expression network analysis (WGCNA)

The R package “WGCNA” was used to identify the highly connected modules and genes, and the soft threshold power *β* was calculated by the pickSoftThreshold function when constructing each module. This method provides power values ranging from 1 to 20 and chooses the power value closest to 0.9 based on the mean connectivity and the scale‐free topological model fitting results. After setting the soft threshold power, the co‐expression module can be constructed using the WGCNA algorithm in R software. The algorithm converts the adjacency information topological overlap through overlap measure gene network connectivity, and by using hierarchical clustering function according to the topological overlap matrix (TOM) spectrum similarity will not express a similar gene is divided into modules. The minimum size of a gene dendrogram was set to 50. Modules with high similarity were aggregated and merged at a height cutoff of 0.25.

### 2.5 Differentially expressed genes (DEGs) analysis

An adjusted *p* < 0.05 plus fold change >2 was used as the cut‐off for significantly differentially expressed genes (DEGs). The “Pheatmap” and “ggplot2” R packages were applied to construct heat maps and volcano maps of DEGs, respectively. The online website (http://www.ehbio.com/test/venn/#/) was used to obtain the intersection of genes of DEGs, WGCNA and GSEA, and intersection gene Venn plot.

### 2.6 Comparison of diagnostic model constructed by random forest (RF) algorithm and support vector machine (SVM)

This study analyzed these two models (RF and SVM) using the explanatory features of the R package “DALEX.” The best model was selected by plotting the residual distribution. ROC curves were used to evaluate the diagnostic performance of the two models (using the http://bioinformatics.com.cn/ drawing tools). In this study, the average modeling error rate was calculated for all genes using the R package “randomForest.” The RF model was then constructed, and the Gini coefficient method was used to calculate the gene importance values. Genes with an importance value greater than 4 were selected to construct the model.

### 2.7 Characteristics of the infiltration of immune cells in the samples

The CIBERSORT algorithm was used to evaluate immune cell infiltration in CRSwNP and healthy control tissues. This algorithm transformed the normalized gene expression matrix into the composition of infiltrating immune cells. The feature matrix defined 22 infiltrating immune cell components, including B cells naïve, B cells memory, Plasma cells, T cells CD8, T cells CD4 naïve, T cells CD4 memory resting, T cells CD4 memory activated, T cells follicular helper, T cells regulatory (Tregs), T cells gamma delta, NK cells resting, NK cells activated, Monocytes, Macrophages M0, Macrophages M1, Macrophages M2, Dendritic cells resting, Dendritic cells activated, Mast cells resting, Mast cells activated, Eosinophils, and Neutrophils.

### 2.8 Acquisition of single‐cell RNA sequencing (scRNA‐seq) data and analysis of sample immune landscape

The scRNA‐seq data were obtained from the GEO database. By searching the GEO database with the keywords (chronic[All Fields] AND rhinosinusitis[All Fields] AND (‘nasal polyps’[MeSH Terms] OR nasal polyps[All] Fields])) AND ((‘single person’[MeSH Terms] OR single[All Fields]) AND (‘cells’[MeSH Terms] OR cell[All Fields]))) AND ‘*Homo sapiens’*[porgn], GSE156285 was retrieved, and this study utilized one healthy control nasal mucosa sample and one nasal polyps sample from this dataset. In addition, an externally validated dataset from the Genome Sequence Archive (No. HRA000772), which consisted of 5 healthy control samples and 11 CRSwNP samples, was used for HMOX1 expression.

### 2.9 Immunofluorescence staining

Nasal mucosa samples from healthy controls (*n* = 6) and nasal mucosa samples from Eos‐CRSwNP (*n* = 6) and NEos‐CRSwNP (*n* = 6) were collected and immunofluorescence staining was performed. DAPI, CD163 antibody (proteintech, 16646‐1‐AP) and HMOX1 antibody (proteintech, 10701‐1‐AP) were used to stain the cell components. Differences in protein staining in each group were observed using an Olympus confocal microscope.

### 2.10 Bone marrow‐derived macrophage (BMDM) differentiation

BMDMs were isolated from the hind limbs of mice, and cultured in DMEM/F12 high‐glucose medium (containing penicillin‐streptomycin) with 50 ng/mL M‐CSF Protein (MCE, HY‐P7085) for 7 days. On Day 7, BMDMs were divided into different groups: control group, IL‐4 group (20 ng/ML), IFN‐γ group (50 ng/mL), and IL‐4+zinc protoporphyrin (ZNPP, HMOX1 inhibitor; MCE, HY‐101193) group. Cells in the IL‐4+ZNPP group were pre‐treated with 25 μM ZNPP for 2 h. At the endpoint of stimulation, cells were stained for membrane markers using 5 μL/million cells of CD11b (Biolegend, 101228), F4/80 (Biolegend, 123120), CD86 (Invitrogen, 1993631) and CD163 (Biolegend, 156704). Cell membranes were then fixed overnight using the Staining Buffer Set (Invitrogen, 00‐5523‐00). After fixation, cells were permeabilized using the Staining Buffer Set (Invitrogen, 00‐5523‐00), followed by intracellular staining for Heme Oxygenase 1 (Abcam, ab237268). Analysis was performed using an Attune NxT flow cytometer.

### 2.11 Statistical analysis

SPSS 26.0 statistical software (IBM SPSS) and GraphPad Prism9.0 (GraphPad Software) were used for statistical analysis. The values were expressed as the mean ± standard error of the mean (SEM). Correlation analysis was performed using the Spearman method. Statistical differences between more than two groups of patients were assessed using one‐way analysis of variance (ANOVA) and the Brown–Forsythe test. A value of *p* < 0.05 was considered statistically significant. Univariate and multivariate analyses were performed using the enter method in unconditional binary logistic regression, and their 95% confidence intervals (CIs) were estimated through the logistic regression models.

## 3 RESULTS

### 3.1 Identification of hub‐DEGs related to the key pathway of CRSwNP

**FIGURE 1:**
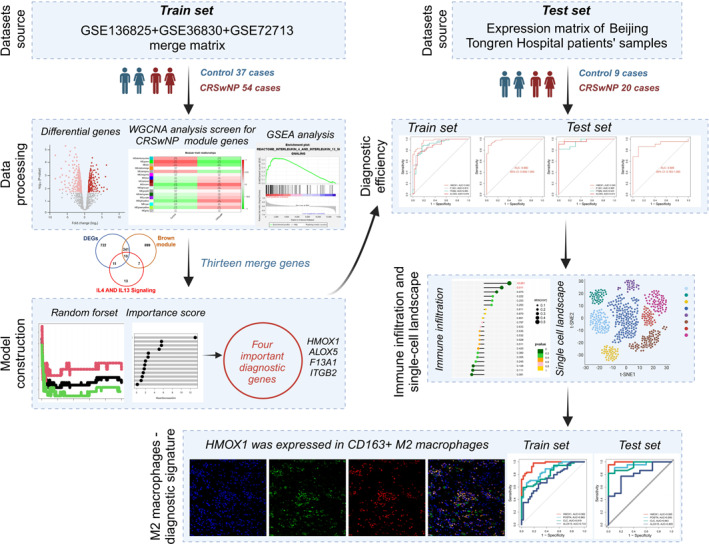
The flow chart of this study. The flowchart was generated by Biorender. Agreement number: KA277W2E4U.

**FIGURE 2:**
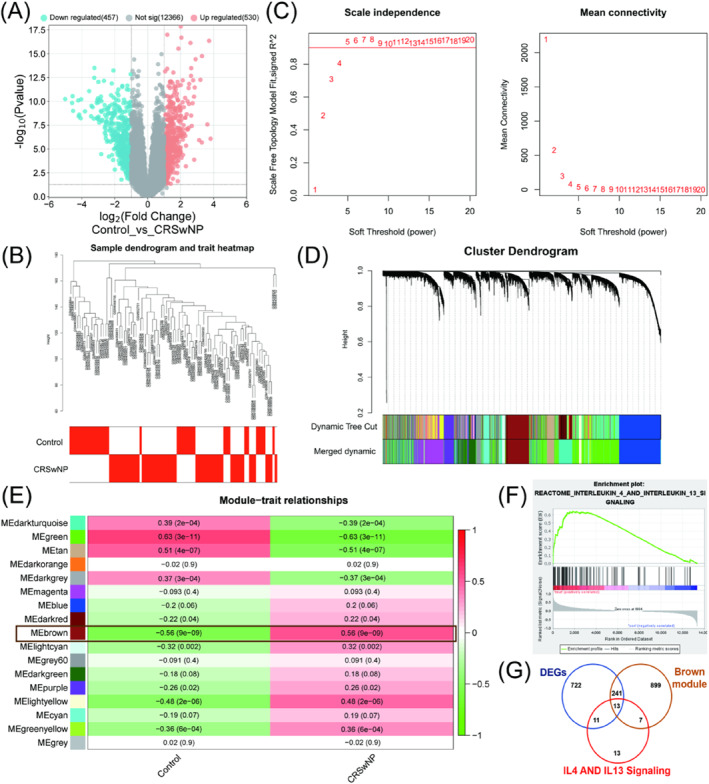
Identification of intersection genes from DEGs, GSEA, and WGCNA analyses. (A) Volcano map of differentially expressed genes between control samples and CRSwNP samples. (B) Dendrogram of the samples clustered with feature heatmap. (C) Scale independence and mean connectivity for multiple soft threshold power (*β*) from 1 to 20. (D) Establishment of co‐expression gene module based on hierarchical clustering algorithm. (E) Correlations between different modules and control samples and CRSwNP samples. (F) The differentially expressed genes between groups are enriched in INTERLEUKIN_4 AND INTERLEUKIN_13 SIGNALING. (G) Venn plot of intersection genes of DEGs, GSEA and WGCNA.

The technical route flow chart of this study is shown in Figure 1. DEGs analysis showed that 530 genes were up‐regulated and 457 genes were down‐regulated in the CRSwNP group (Figure 2A). WGCNA was applied to explore the potential functions of DEGs, and construction of a hierarchical clustering tree demonstrated 17 modules, each of which was labeled with a distinct color. MEbrown module (*r* = 0.56, *p* < 0.001) showed the most significant elevation in the CRSwNP group (Figure 2B–E). Genes of this module were then performed with GSEA analysis, which indicated that DEGs in the CRSwNP group were predominantly enriched in the INTERLEUKIN_4_AND_INTERLEUKIN_13_SIGNALING pathway (Figure 2F). Intersection analysis among genes of WGCNA, GSEA, and up‐regulated DEGs in CRSwNP identified 13 hub‐genes, including HMOX1, F13A1, ITGB2, ITGAM, ALOX5, ITGAX, CCL11, IL10, MMP9, IGHG1, IL1B, OSM, and FCER2, as shown in Figure 2G.

### 3.2 The diagnostic model constructed by RF algorithm

**FIGURE 3:**
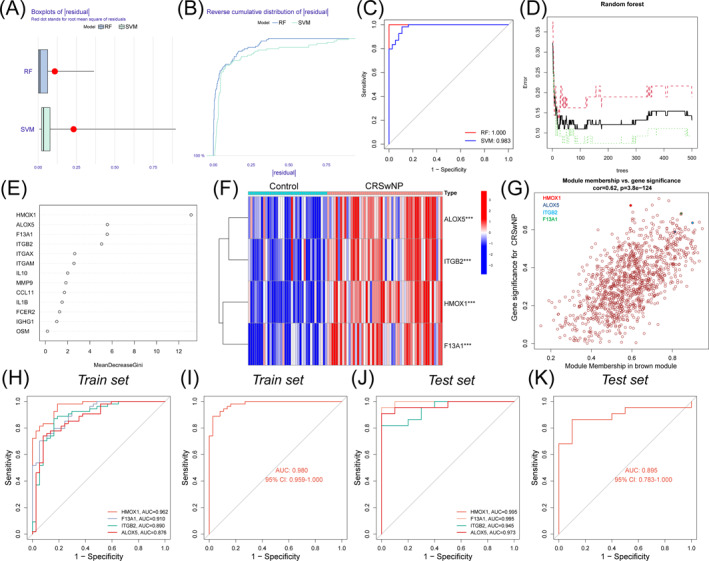
Construction and evaluation of diagnostic models for CRSwNP. (A) Block diagram of sample residuals. The red dot represents the root mean square of the residual. (B) Cumulative residual distribution of samples. (C) ROC curve and AUC value of RF model and SVM model. (D) Error decision tree: the green curve represented control error, the red curve represented the error in determining CRSwNP, and black represented error for all samples. (E) Relative importance of explanatory variables ranked by RF model. (F) Expression heatmap of seven diagnostic hub genes in control samples and CRSwNP samples. (G) Scatter plot shows the relationship between module membership and gene significance in the MEbrown module. (H‐I) ROC curves of the independent and integrated diagnostic efficacy of seven diagnostic genes for CRSwNP in the train set. (J‐K) ROC curves of the independent and integrated diagnostic efficacy of seven diagnostic genes for CRSwNP in the test set.

To evaluate the effectiveness of different algorithms in constructing diagnostic models, we compared the diagnostic performance of the RF algorithm and the Support Vector Machine (SVM) algorithm. The ROC curve analysis revealed that the RF model demonstrated superior diagnostic performance with an AUC of 1.000 compared to the SVM model, which had an AUC of 0.983. Additionally, RF had the fewer sample residuals (Figure 3A–D). The results showed that four genes (HMOX1, ALOX5, F13A1 and ITGB2) had an importance score >4 (Figure 3E),^17^, ^18^, ^19^, ^20^, ^21^ which were further used as the diagnostic gene set for CRSwNP. The basic characteristics and functions of the four genes are presented in Table S2. The heatmap showed that all the seven genes were significantly upregulated in the CRSwNP group (*p* < 0.001; Figure 3F). The correlations of gene module membership in MEbrown and gene significance for CRSwNP are shown in Figure 3G, indicating that the seven diagnostic genes were highly correlated with its corresponding module and its corresponding traits (CRSwNP). The ROC curve of the training set showed that the AUC value of each single gene was all greater than 0.8, and the combined AUC value of seven genes was 0.980 (95% CI: 0.959–1), indicating their good diagnostic efficacy for CRSwNP (Figure 3H,I). In addition, these diagnostic genes also showed excellent diagnostic efficacy in the test dataset (AUC = 0.895; 95% CI: 0.783–1; Figure 3J,K).

### 3.3 The increased infiltration of M2 macrophages and HMOX1 expression in CRSwNP

**FIGURE 4:**
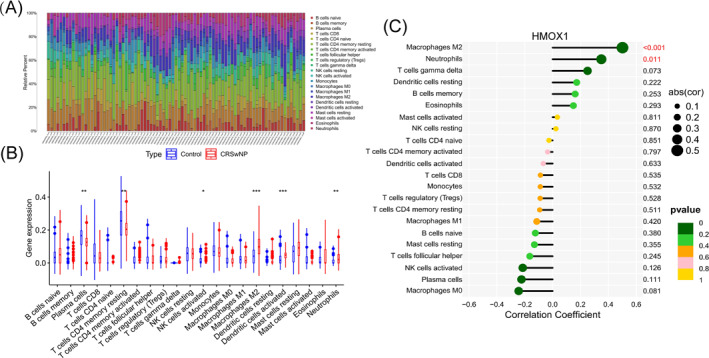
The infiltration of 22 immune cells in the control group and CRSwNP group, and the correlation between diagnostic genes and immune cell infiltration. (A) The proportion of 22 types of immune cells in each sample. (B) Differences in the degree of different types of immune cell infiltration between control and CRSwNP samples. (C) Correlation between the expression of HMOX1 and infiltration of different types of immune cells.

Immune infiltration analysis revealed significant differences in the infiltration of immune cells between the control group and the CRSwNP group. There was an increased infiltration level of activated NK cells, M2 macrophages, activated dendritic cells, and neutrophils in CRSwNP compared to the control group. Notably, M2 macrophage infiltration showed the most significant difference (*p* < 0.001; Figure 4A,B). Additionally, a strong positive correlation was observed between HMOX1 expression and M2 macrophage infiltration (*p* < 0.001; Figure 4C).

**FIGURE 5:**
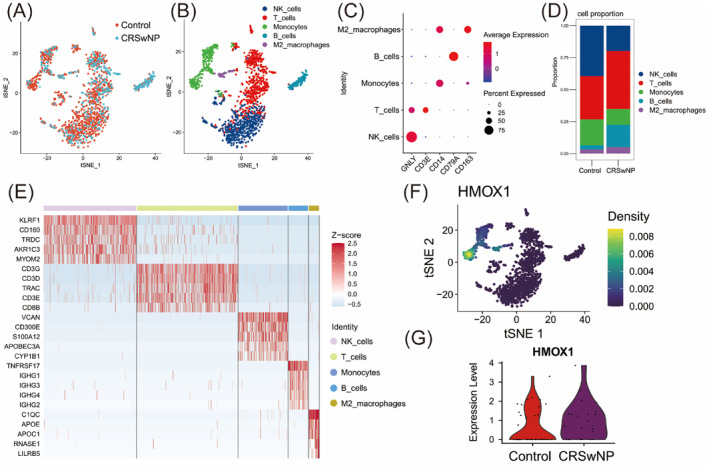
Single‐cell landscape of immune cells in nasal mucosa of CRSwNP. (A‐B) T‐Stochastic neighbor embedding (tSNE) plots showed immune cells from the control and CRSwNP samples. (C) Bubble map of annotated genes for each cell subset. (D) Proportion of all cell subsets in the Control sample and the CRSwNP sample. (E) Top five expression genes heatmap of all cell subsets. (F) T‐SNE plots of the distribution of HMOX1 in all cell subsets. (G) Violin plot of the expression level of HMOX1 in M2 macrophages.

Based on the analysis of scRNA‐seq data from nasal mucosa, the immune cells in the samples were classified into five subgroups and annotated as follows: NK_cells (GNLY), T_cells (CD3E), Monocytes (CD14), B_cells (CD79A), and M2 macrophages (CD163) (Figure 5A–C). The proportion of T_cells, B_cells and M2_ macrophages increased in the CRSwNP sample, while NK_cells and Monocytes decreased in the CRSwNP sample (Figure 5D). The top 5 genes with the highest expression levels in each cell subset are displayed in Figure 5E. Analysis of HMOX1 expression at the single cell level showed that HMOX1 was mainly distributed in M2_macrophages and Monocytes (Figure 5F; Figure S1A–D). Compared with M2_macrophages of the control sample, the expression of HMOX1 was increased in M2_macrophages of the CRSwNP sample but not in M1_macrophages (Figure 5G; Figure S1E).

### 3.4 HMOX1 was highly expressed in CD163+ M2 macrophages of CRSwNP and positively correlated with eotaxin genes; inhibition of HMOX1 expression reduced CD163+ M2 macrophages

**FIGURE 6:**
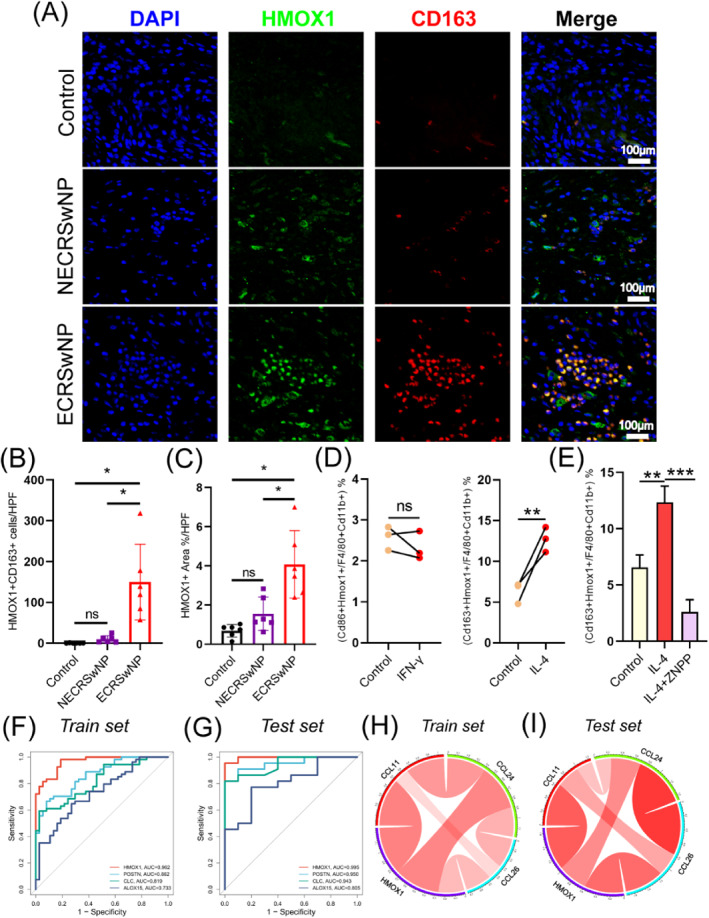
Relationship between HMOX1 and M2 macrophages. (A) Immunofluorescence staining: HMOX1 is marked in green, M2 macrophage marker CD163 in red, and nuclei in blue. (B) Statistical analysis of the number of HMOX1+CD163+ M2 macrophages in samples under High Power Field (HPF). (C) Statistical analysis of the proportion of HMOX1+ area in samples under HPF. (D) Flow analysis of cells after treatment with IFN‐γ or IL‐4 in the mouse macrophage differentiation model. (E) Flow analysis of cells after treatment with HMOX1 inhibitor ZNPP in the M2 macrophage differentiation model. (F‐G) ROC curve for comparison of HMOX1 with other diagnostic biomarkers in the train set and test set. (H‐I) The expression of HMOX1 is positively correlated with the expression of eotaxin genes in the train set and test set.

Immunofluorescence staining demonstrated that HMOX1 and CD163 were co‐labeled in the cells. Compared to healthy controls and non‐eosinophilic (NE) CRSwNP, eosinophilic (E) CRSwNP samples showed a marked increase in HMOX1+CD163+ macrophages (Figure 6A–C), highlighting a close association between HMOX1 and CD163+ M2 macrophages. The results of BMDM differentiation experiments showed that IFN‐γ stimulation did not change the proportion of CD86+HMOX1 M1 macrophages, while IL‐4 promoted M2 macrophage polarization (Figure 6D; Figure S2). On the contrary, ZNPP treatment significantly reduced the proportion of CD163+HMOX1+ M2 macrophages (*p* < 0.001) (Figure 6E), which suggests that HMOX1 may regulate M2 macrophage polarization.

In both the training set and the test set, HMOX1 had similar diagnostic performance for CRSwNP compared to other well‐known biomarkers such as POSTN, CLC and ALOX15 (Figure 6F,G).^22^, ^23^, ^24^ Correlation analysis showed that HMOX1 expression was positively correlated with the eotaxin genes CCL11, CCL24 and CCL26 in the training set and test set (*p* < 0.05; Figure 6H,I). These findings demonstrated a close connection between HMOX1 and M2 macrophages, and suggested their potential role in regulating eosinophilic inflammation.

## 4 DISCUSSION

CRSwNP is primarily associated with type 2 inflammation characterized by a significant presence of eosinophils.^25^, ^26^ Macrophages play an important role in the pathology of this disease, and exhibit remarkable plasticity which differentiated into two key subsets. M1 macrophages are pro‐inflammatory and polarized by lipopolysaccharide alone or in combination with Th1 cytokines such as IFN‐γ and GM‐CSF, producing pro‐inflammatory cytokines including IL‐1β, IL‐6, IL‐12, IL‐23 and TNF‐α. Alternatively, M2 macrophages are traditionally viewed as anti‐inflammatory and immunomodulatory cells, and polarized by Th2 cytokines IL‐4 and IL‐13, leading them to produce anti‐inflammatory cytokines like IL‐10 and TGF‐β.^27^ However, the roles of M2 macrophages appear to be more complex in CRSwNP. M2 macrophages also have the ability to recruit eosinophils and contribute to tissue remodeling. In addition, the role of IL‐10 is far more complex and may affect epithelial function and be involved in allergies.^28^ Recently, Ma et al. reported a subpopulation of CD109^+^ Th2 cells that can produce IL‐10, which may partially explain the upregulation of IL‐10 in CRSwNP.^29^ Besides, the production of TGF‐β may also contribute to tissue remodeling and fibrosis, perpetuating the inflammation. Recent findings provide strong evidence for the increased infiltration of M2 macrophages in CRSwNP.^30^, ^31^ However, the role of M2 macrophages in CRSwNP needs further investigation.

This study established a link between genes in the diagnostic model and M2 macrophages. All the genes in the diagnostic model have good diagnostic efficacy for CRSwNP. Immune infiltration analysis showed that HMOX1 was significantly positively correlated with M2 macrophage infiltration. What is more, immunofluorescence staining results showed that HMOX1 was co‐expressed with CD163+ M2 macrophages and was significantly overexpressed in CRSwNP. Correlation analysis showed that HMOX1 expression was positively correlated with eotaxins (CCL11, CCL24, CCL26), suggesting a potential association of HMOX1 with eosinophilic inflammation. In addition, HMOX1 had a similar diagnostic performance to POSTN, CLC and ALOX15, which were also identified as diagnostic markers for CRSwNP. Interestingly, when asthma was used as a disease control for CRSwNP, the expression of HMOX1 in the nasal mucosa of asthma patients was comparable compared to that of healthy controls (Figure S3).^32^ Finally, we concluded that HMOX1+ M2 macrophages could be used as a novel high performance diagnostic signature for CRSwNP.

HMOX1 has been studied extensively in a variety of diseases. It catalyzes the degradation of heme into carbon monoxide, ferrous iron, and bilverdin, which is subsequently converted to bilirubin. Over the past 2 decades, carbon monoxide and biliverdin/bilirubin have been shown to modulate key cellular processes such as inflammation, cell proliferation, and apoptosis as well as antioxidant defenses.^33^, ^34^, ^35^ It has been reported that HMOX1, as a heat shock protein induced by hepatic ischemia‐reperfusion injury (IRI) stress, activated the intracellular SIRT1/autophagy axis to alleviate IRI in orthotopic liver transplantation.^36^ Devendra et al. hypothesized that HMOX1 might protect the body from COVID‐19 induced inflammation as well as acute respiratory distress syndrome, and coagulopathy.^37^ Our findings demonstrated that HMOX1 was expressed in macrophages and co‐labeled with CD163. Compared to healthy controls, CRSwNP showed a marked increase in HMOX1+ CD163+ macrophages. It has been shown that macrophages from wild‐type mice could restore heme circulation in Hmox1^−/−^ mice and although the effect is transient, they could reverse anemia and intravascular hemolysis, normalize blood chemistry and iron metabolism parameters, and prevent renal damage.^38^ Furthermore, binding of hemoglobin:haptoglobin complexes to CD163‐expressing macrophages induces potent IL‐10 secretion, which in turn induces HMOX1 stress protein synthesis through an autocrine mechanism. Those findings indicated anti‐inflammatory roles of HMOX1 in macrophages, which may be relevant for atheroprotection, wound healing, and postoperative patient recovery.^39^ However, the role of HMOX1 in the pathogenesis of CRSwNP is still unclear.

Our findings show that HMOX1 expression is positively correlated with multiple eotaxins. Interestingly, a study found that sputum TIPE2 levels were significantly increased in patients with eosinophilic asthma and significantly decreased in patients with neutrophilic asthma. Abnormal expression of TIPE2 may inhibit M1 macrophage‐associated neutrophilic inflammation in asthma by targeting the Nrf2/HMOX1 pathway.^40^ Notably, in an OVA‐induced mouse model of eosinophilic asthma, HMOX1 induction suppressed Th2 responses and reduced apoptosis of primary airway epithelial cells.^41^ However, Han et al. pointed out that excessive activation of M2 macrophages in turn aggravates‐allergic asthma.^42^ Taken together, HMOX1 may play a 'double‐edged sword' role in airway inflammation.

This study screened candidate diagnostic genes by combining DEGs analysis, GSEA analysis, immune infiltration analysis and WGCNA, and further constructed a diagnostic model using a RF algorithm. The relationship between the diagnostic genes and macrophages was further verified from multiple dimensions by transcriptome data, single‐cell sequencing data, immunofluorescence and correlation analysis. Finally, we found that the expression of HMOX1 was positively correlated with the infiltration of M2 macrophages and highly expressed in CD163+ M2 macrophages in the CRSwNP group, indicating its superior diagnostic value for CRSwNP. However, this study is somewhat limited: First, although we showed that the increased expression of HMOX1 was significantly correlated with CRSwNP, the clinical significance remains limited due to lack of functional data on the role of HMOX1 in the pathogenesis of CRSwNP. Second, public datasets included in this study lack more detailed clinical information to confirm the diagnostic value of HMOX1 in distinguishing ECRSwNP and NECRSwNP. Third, the relationship between HMOX1 and M2 macrophage, and their roles in CRSwNP is unclear, which needs further investigations.

## 5 CONCLUSION

M2 macrophage‐derived HMOX1 can be used as an innovative diagnostic signature for CRSwNP, which might be a potential regulator of eosinophilic inflammation.

## AUTHOR CONTRIBUTIONS

**Enhao Wang**: Investigation; writing—original draft; writing—review and editing; formal analysis; methodology. **Yanghe Hao**: Data curation; methodology; investigation. **Jing Song**: Investigation; formal analysis; data curation. **Jing Yuan**: Investigation; formal analysis; data curation. **Yu Hong**: Investigation; formal analysis. **Ying Li**: Investigation. **Yang Wang**: Investigation. **Chengshuo Wang**: Conceptualization; writing—review and editing; project administration. **Ming Wang**: Conceptualization; investigation; funding acquisition; writing—review and editing; writing—original draft; project administration. **Luo Zhang**: Conceptualization; funding acquisition; writing—original draft; writing—review and editing; project administration.

## CONFLICT OF INTEREST STATEMENT

The authors declare that they have no conflicts of interest.

## Supporting information

Supporting Information S1
