# Supplementary material for: M2 macrophage derived HMOX1 defines chronic rhinosinusitis with nasal polyps
Source: Clin Transl Allergy. 2024 Dec 7;14(12):e70014. doi: 10.1002/clt2.70014 (PMC11624889; doi:10.1002/clt2.70014)
Supplement: Supplementary file 1 — Supporting Information S1 [file CLT2-14-e70014-s001.docx]

**Supplementary figures and tables**

**Figure S1** HMOX1 expression in different cell types of CRSwNP. (A) Uniform manifold approximation and projection (UMAP) showing the identification of 13 cell clusters in nasal mucosa samples. Data obtained from Genome Sequence Archive (No: HRA000772). (B) Expression of HMOX1 in all cell subpopulations. (C) The distribution of M1 and M2 macrophages. (D) Expression of HMOX1 in M1 and M2 macrophages. (E) Differential analysis of HMOX1 expression levels in M1 and M2 macrophages from CRSwNP and control groups.


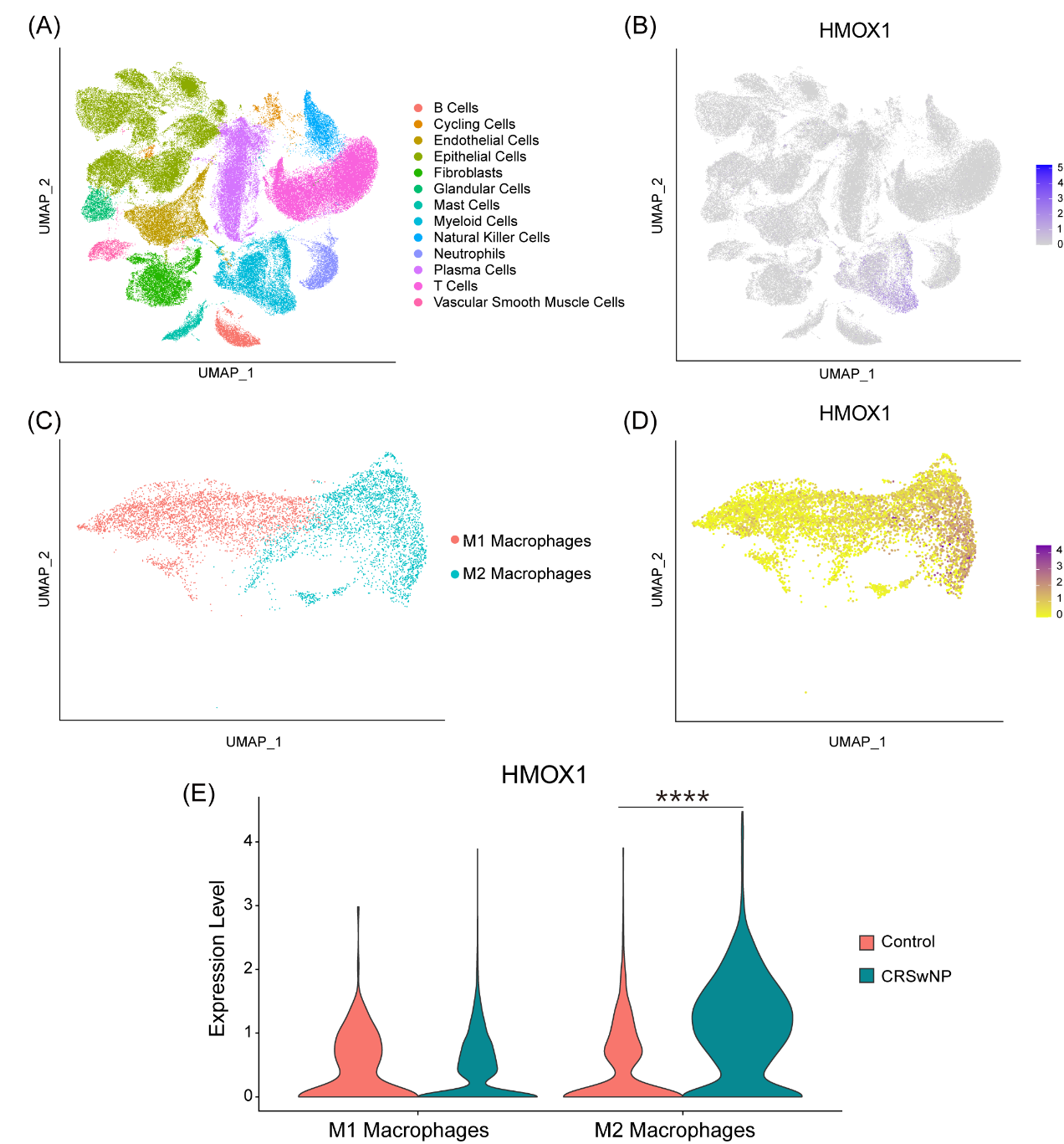


**Figure S2** Diagram of the process of mouse macrophage differentiation model.


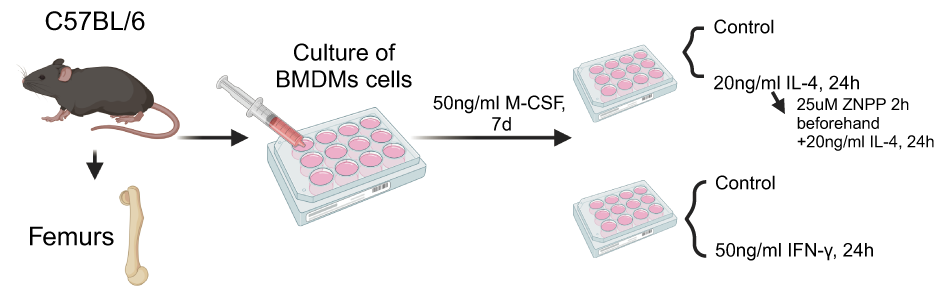


**Figure S3** Expression of HMOX1 in nasal mucosa samples of asthma and control subjects. Transcriptome data are from Synape platform (syn9878922) and GEO dataset (GSE41861).


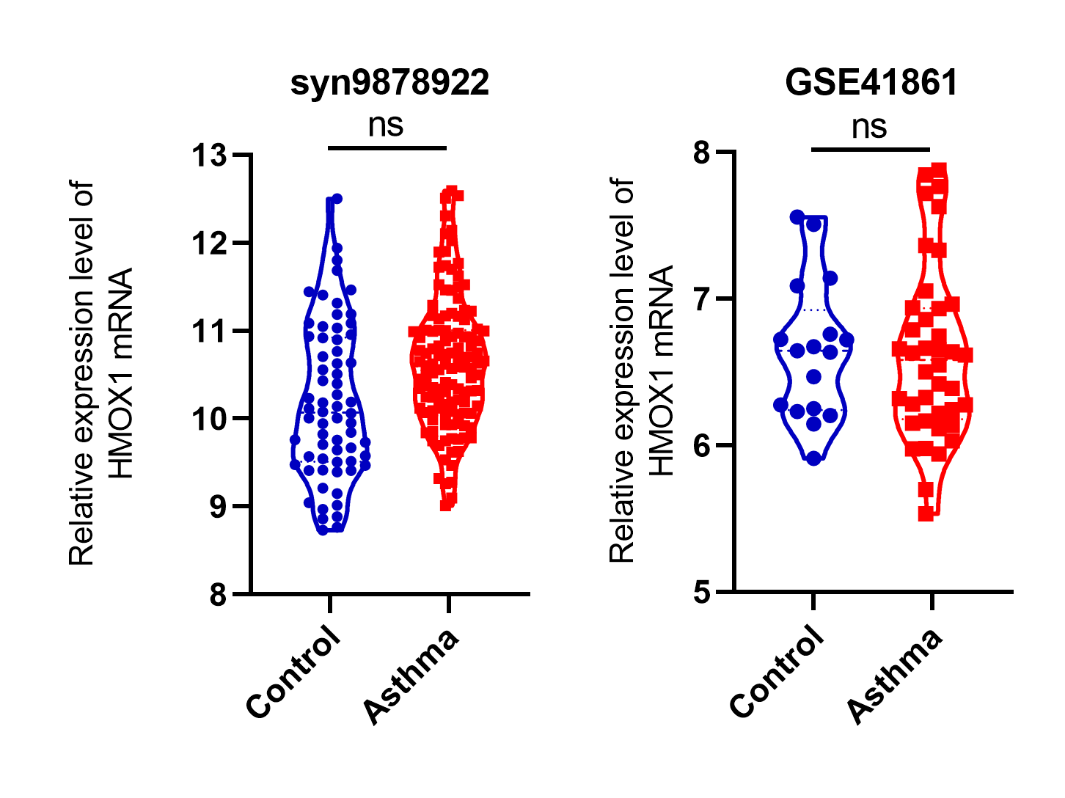


**Table S1** Summary information for RNA sequencing data used in this study.

| **Dataset** | **References** | **Control**  **(cases)** | **CRSwNP**  **(cases)** | **Applications** |
| --- | --- | --- | --- | --- |
| GSE136825 | PY, et al, Eur Respir J (2019) | 28 | 42 | Training set |
| GSE36830 | SS, et al, Allergy (2012) | 6 | 6 | Training set |
| GSE72713 | WW, et al, Sci Rep (2016) | 3 | 6 | Training set |
| TongRen | WM, et al, Clin Transl Allergy (2020) | 9 | 20 | Test set |

**Table S2** Summary information of the four diagnostic genes.

| **Gene** | **Full name** | **Functions** | **Related diseases** | **Citation** |
| --- | --- | --- | --- | --- |
| HMOX1 | Heme oxygenase 1 | The enzymatic reaction catalyzed by HMOX1 yields Fe2+, biliverdin, and CO. HMOX1 and the by-product CO can down-regulate destructive immune responses in several models of intestinal inflammation | Inflammatory bowel diseases, including Crohn's disease and ulcerative colitis | ^17^ |
| F13A1 | Factor XIII subunit A | F13A1 is not only involved in coagulation, but may also play a role in wound healing and basic immune functions | Wound healing, chronic inflammatory bowel disease, arteriosclerosis, rheumatoid arthritis, chronic inflammatory lung disease, chronic sinusitis, solid tumors, hematologic malignancies, and obesity | ^18^ |
| ITGB2 | Integrin subunit beta 2 | ITGB2, dimerize with each other to form integrin αMβ2, called macrophage-1 antigen (Mac-1). Mac-1 is expressed in many types of leukocytes, especially in eosinophils. Mac-1 plays a key role in innate and adaptive immune responses | Chronic rhinosinusitis, Alzheimer's disease, type 2 diabetes | ^19^ |
| ALOX5 | Arachidonate 5 - lipoxygenase | ALOX5 plays a central role in cell death, including apoptosis, pyroptosis, and ferroptosis. ALOX5 is required for the recruitment of eosinophils in the peritoneal cavity | Colorectal cancer, breast cancer, glioma and other inflammation-related cancers | ^20-21^ |
